# Dataset of temporal trends of surface water area across India's rivers and basins

**DOI:** 10.1016/j.dib.2023.109991

**Published:** 2023-12-19

**Authors:** Pradeep Koulgi, Suman Jumani

**Affiliations:** a Independent Researcher, Bangalore, India; b Foundation for Ecological Research, Advocacy and Learning, Bangalore, India

**Keywords:** Water extent, Water availability, Drought, Google Earth Engine

## Abstract

This dataset [1] quantifies the extent and rate of annual change in surface water area (SWA) across India's rivers and basins over a period of 30 years spanning 1991 to 2020. This data has been derived from the Global Surface Water Explorer, which maps historical terrestrial surface water occurrence globally using the Landsat satellite image archive since 1984, at a spatial resolution of 30 m/pixel and a temporal resolution of once a month. This monthly time-series was used to create annual composites of wet-season (October, November, December), dry-season (February, March, April), and permanent (October, November, December, February, March, April) surface water extent, which were then used to estimate annual rates of change. To estimate SWA trends for both river networks and their basins, we conducted our analysis at two spatial scales – (1) cross-sectional reaches (transects) across river networks, and (2) sub-basins within river catchments. For each reach and sub-basin (henceforth basin), temporal trends in wet-season, dry-season, and permanent SWA were estimated using the non-parametric Sen's slope estimator. For every valid reach and basin, the temporal timeseries of invalid or missing data was also computed as a fractional area to inform the level of certainty associated with reported SWA trends estimates.

In addition to a Zenodo data repository, this data [1] is presented as an interactive web application (https://sites.google.com/view/surface-water-trends-india/; henceforth Website) to allow users to visualize the trends of permanent, wet-season, and dry-season water along with the extent of missing data for individual transects or basins across India. The Website provides a simple user interface to enable users to download seasonal time-series of SWA for any region of interest at the scale of the river network or basin. The Website also provides details about accessing the annual permanent, dry and wet season composites, which are stored as publicly accessible cloud assets on the Google Earth Engine platform. The spatial (basin and reach) and temporal (wet season, dry season, and permanent water scenarios) scales of information provided in this dataset yield a granular understanding of water systems in India. We envision this dataset to serve as a baseline information layer that can be used in combination with other data sources to support regional analysis of hydrologic trends, watershed-based analysis, and conservation planning. Specific applications include, but are not limited to, monitoring and identifying at-risk wetlands, visualizing and measuring changes to surface water extent before and after water infrastructure projects (such as dams and water abstraction projects), mapping drought prone regions, and mapping natural and anthropogenic changes to SWA along river networks. Intended users include, but are not limited to, students, academics, decision-makers, planners, policymakers, activists, and others interested in water-related issues.

Specifications Table

**Table:** 

Subject	Global and Planetary Change Hydrology and Water quality
Specific subject area	Trends in surface water area
Data format	Filtered, Analyzed
Type of data	Dataset (table) Shapefile Results graph Interactive website
Data collection	Global Surface Water Explorer data were aggregated at two scales. The pixel-level aggregation combined data for a 3-month period to get a seasonal composite of water, not water, or no data for dry and wet seasons per water year. Seasonal-scale aggregation combined the two seasonal composites of a pixel to get an annual composite. Timeseries data for a basin or reach were considered for trend analysis only if ≥ 95 % its area contained valid data from the pixel-level aggregation. We discarded transects and basins with fewer than 5 valid data points. Finally, temporal trends in seasonal and permanent surface water extent were estimated for every valid transect and basin using Sen's slope estimator.
Data source location	Global Surface Water Explorer Data [2] HydroSHEDS Flow Accumulation Data, at 15” per pixel resolution [4] HydroSHEDS Flow Direction Data, at 15” per pixel resolution [4] HydroBASINS Dataset's level 7 river basins [4]
Data accessibility	Repository name: Temporal trends of surface water data across India's rivers and basins Data identification number: 10.5281/zenodo.7803903 Direct URL to data: http://dx.doi.org/10.5281/zenodo.7803903 Instructions for accessing these data: Download the data from the Zenodo archive above, and refer to its 00_README.txt for details on its attributes. Code repository: https://doi.org/10.5281/zenodo.7839588

## 1 Value of the Data

• The temporal dynamics of SWA at two spatial scales and three seasons across 30 years provides valuable information relevant to several fields including public health, food security, agriculture systems, climate, and conservation.
• This data is useful to students, academics, decision-makers, planners, policymakers, activists, and others interested in water-related issues.
• This data can serve as a baseline information layer that can be used in combination with other data sources to support regional analysis of hydrologic trends, watershed-based analysis, and conservation planning.
• Specific applications can include monitoring and identifying at-risk wetlands (specifically, loss in SWA for seasonal and perennial wetlands, gains in SWA in response to restoration activities etc.), measuring changes to surface water extent before and after water infrastructure projects (such as reservoirs, diversion-type dams or water abstraction projects), mapping drought prone regions, and mapping natural and anthropogenic changes to SWA along river networks.
• This dataset is particularly useful given the general scarcity of accessible and robust comparable national-level datasets in India.

## 2 Background

The main objective of the dataset is to provide an accessible and user-friendly dataset of temporal and seasonal dynamics of surface water extent and trends of change for rivers and basins across India. The creation of a Website further allows users to interactively visualize trends in surface water at various locations and spatial scales across India. Given the relevance of surface water dynamics to climate, water and food security, public health, social and economic wellbeing, biodiversity, and associated ecosystem services, we anticipate this dataset to be useful to several stakeholder groups. Easy visualizations and data download capabilities can be useful for purposes including, but not limited to, exploratory analysis, monitoring, modeling, predicting, and decision-making in various fields.

## 3 Data Description

The dataset is available for interactive visualization and download at https://sites.google.com/view/surface-water-trends-india/ as well as in the Zenodo data repository. This dataset provides a time-series of annual wet-season, dry-season, and permanent SWA for a 30-year period extending from 1991 to 2020 for rivers and basins across India. The Website has three main components – interactive data visualization, open data access, and open methods. Each is described below.

### 3.1 Interactive data visualization

**Fig 1:**
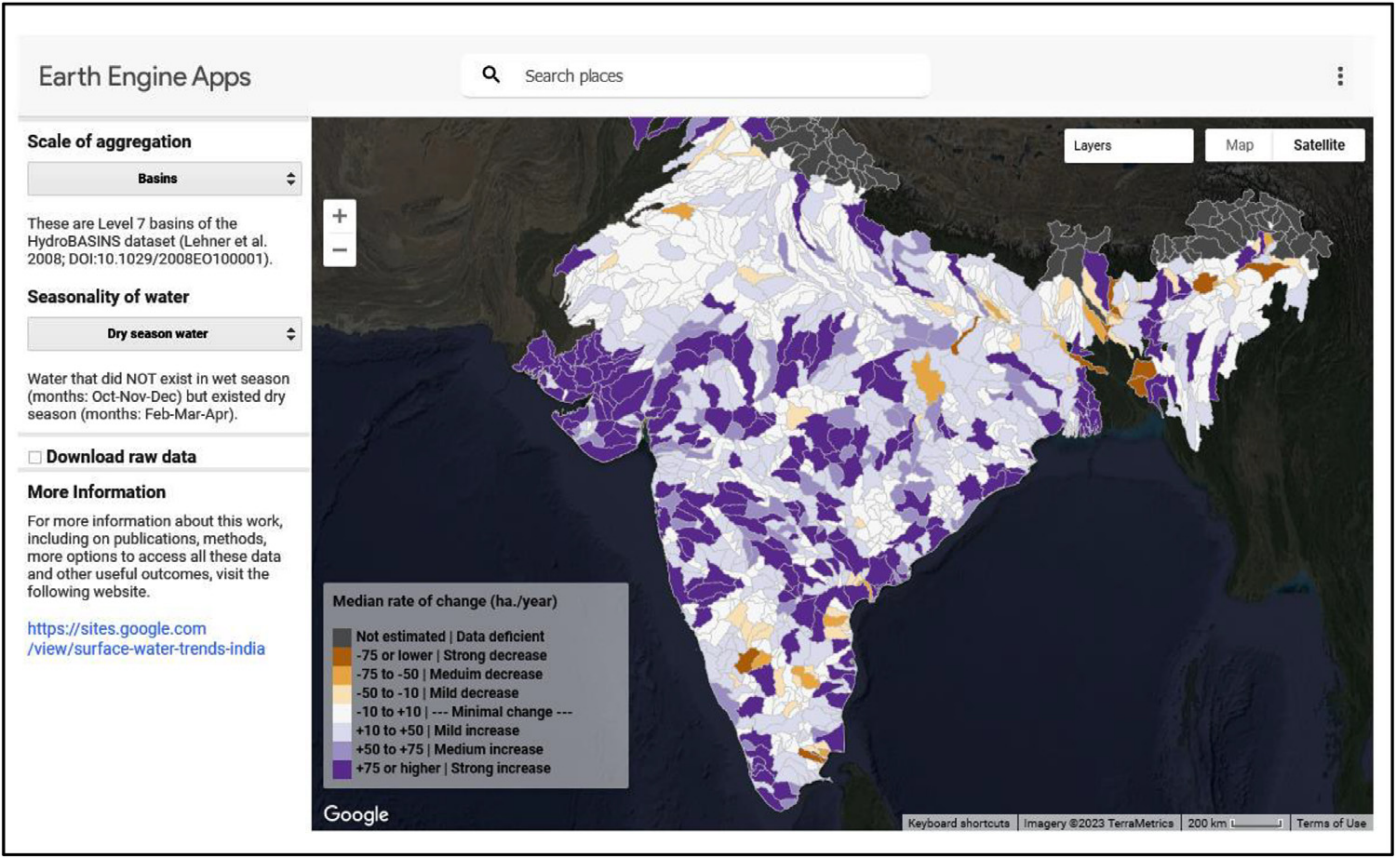
Example display of the interactive data visualization tool.

Users can access the interactive data visualization and download tool by clicking on the ‘Explore Map’ button on the homepage or selecting the ‘Map’ header on the Website. This will open an interactive embedded Google Earth Engine application (Fig 1) where the spatial scale of interest (basins or reaches) and season (dry season water, wet season water, or permanent water) can be selected. Upon selection, each term is defined below. The spatial features are colored across a gradient of eight categories to indicate an increasing (shades of purple) or decreasing (shades of orange) trend of change in SWA as indicated by the legend on the map panel.

**Fig 2:**
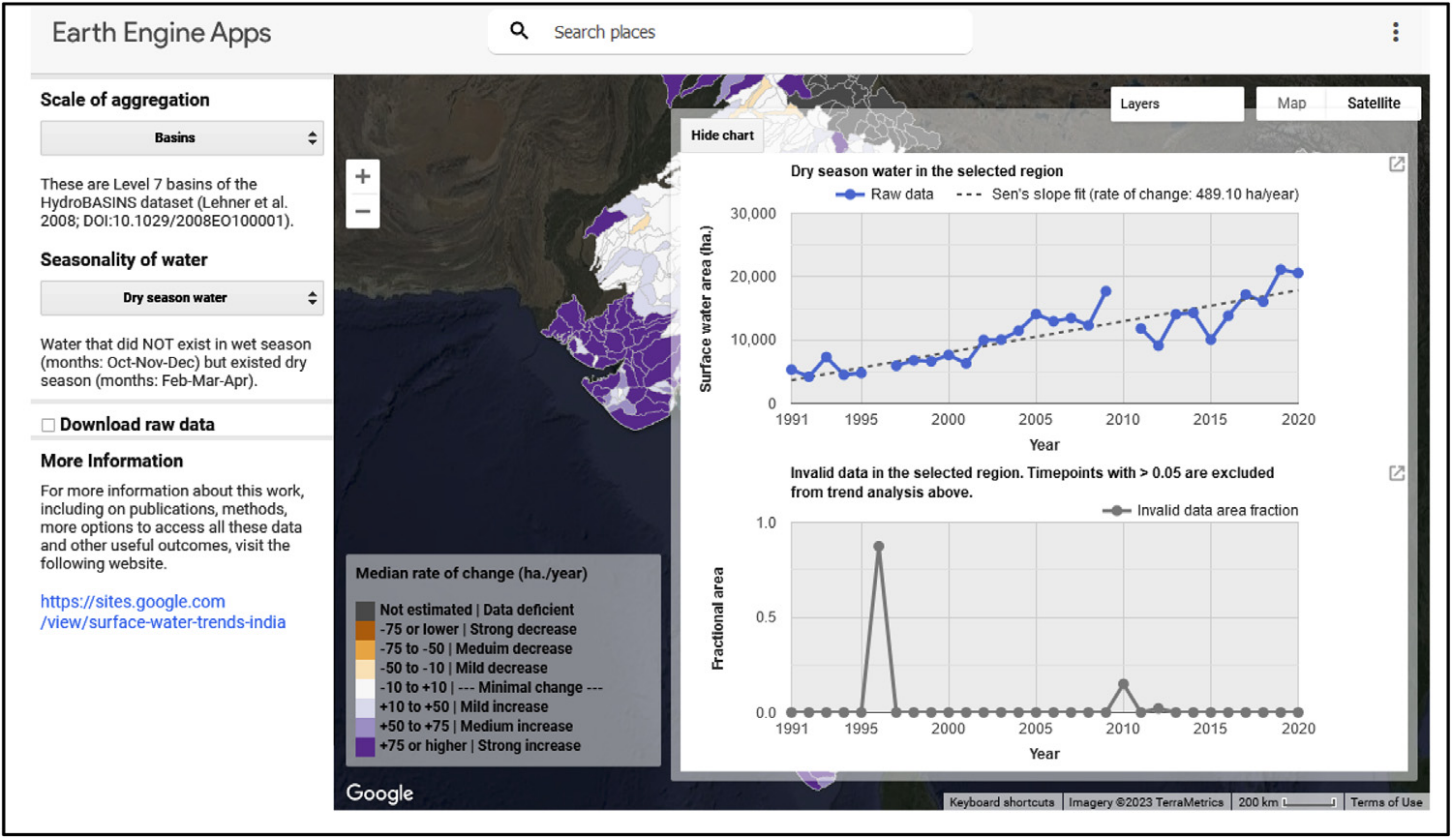
Upper panel: Timeseries of dry-season surface water area for a selected basin (solid blue line) and annual trends of change as computed by a Sen's slope (dashed grey line). Lower panel: Corresponding timeseries of percent no-data (grey solid line).

Users can then click on features of interest (basins or reaches) to visualize the underlying timeseries and annual trends of change in SWA. Upon selection, the feature gets highlighted in green and two figures are generated (Fig 2). The upper panel of the figure illustrates the underlying timeseries of SWA for the feature of interest (solid blue line), annual trends of change as computed by a Sen's slope estimator (dashed grey line), and a slope fit or rate of change (in hectares/year). The lower panel provides corresponding information on the fractional area of invalid data across the time-series to help users infer confidence or certainty associated with reported trends.

Data presented in the Website can be downloaded in CSV (comma-separated values) format by checking the ‘Download raw data’ option on the left panel. Instructions for the user to select a few features of interest and download a CSV for them are listed in the “How to” section of the same Map page as well as in the “Download Data as CSV” webpage accessible from the Website's top menu. The downloaded data table includes data on the extent of surface water and no data across all three seasons for the selected features of interest. The nine columns in the data file include: (1) HYBAS_ID (for basins) or txID (for reaches): a unique identifier for each feature, (2) area_ha: area encompassed by the basin or reach in hectares, (3) year: the year the data pertains to; (4) dry_fma_nodata_ha: The extent of no data in the dry season in hectares, (5) dry_fma_water_ha: The extent of dry season SWA in hectares, (6) wet_ond_nodata_ha: The extent of no data in the wet season in hectares, (7) wet_ond_water_ha: The extent of wet season SWA in hectares, (8) total_nodata_ha: Combined no data extent across all seasons in hectares, and (9) prm_DnW_water_ha: The extent of permanent SWA (i.e. water in wet and dry seasons) in hectares.

The ’How to’ section located below the interactive map also provides detailed instructions on visualizing and downloading data of interest via this map.

### 3.2 Open data access

**Fig 3:**
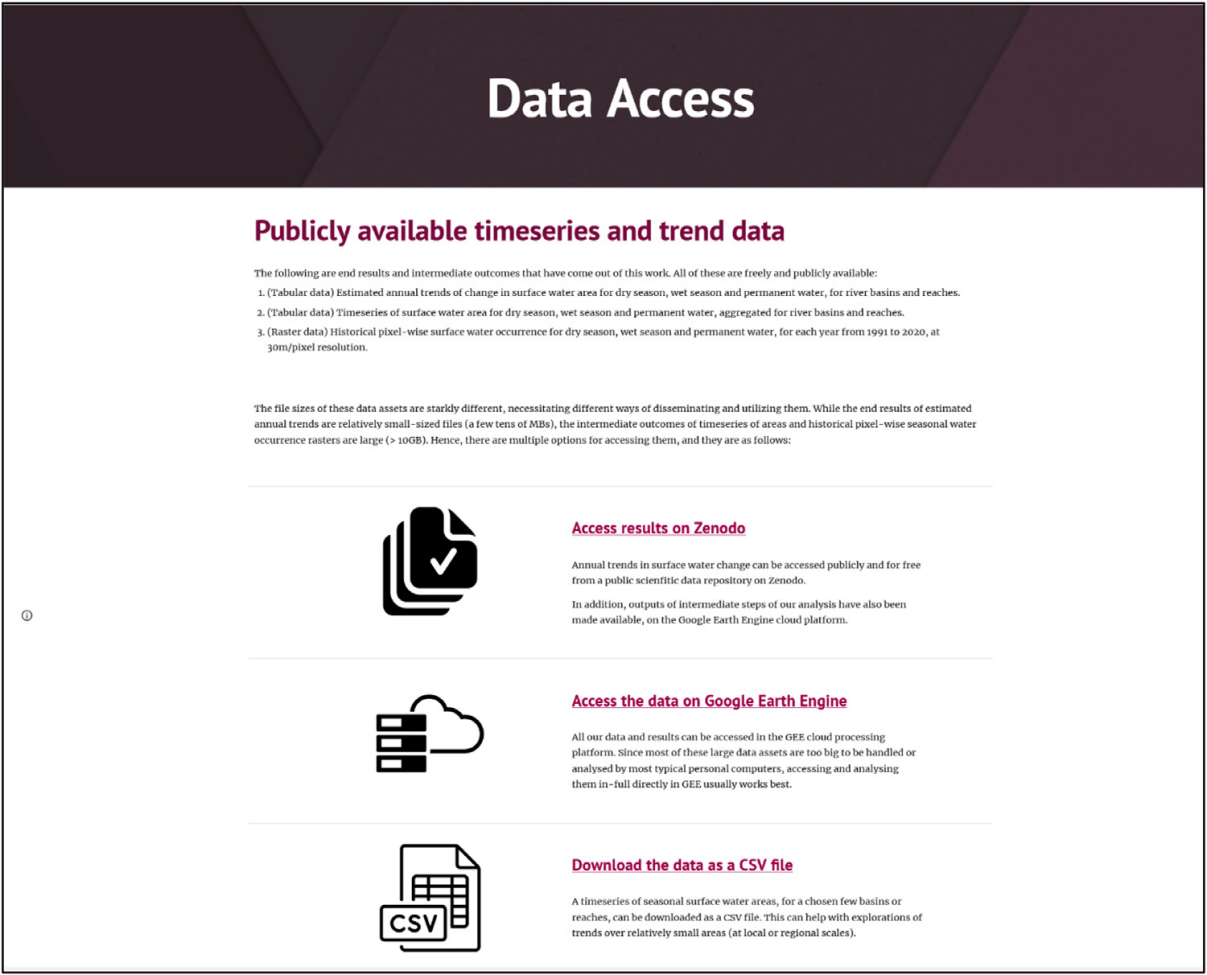
Screenshot of the Open Data Access section from the Website. See Data accessibility in the Specifications table for the Website's link.

Users can navigate to this section by clicking the ‘Open data’ icon on the homepage or selecting the ‘Data Access’ header. This section provides links to access the data via the Zenodo data repository, and annual seasonal composites and other intermediate results as cloud assets on Google Earth Engine, or a subset of them as a CSV via the interactive web map described above (Fig 3).

The data in the Zenodo repository include ESRI Shapefiles, for both basins and reaches, containing the estimated annual trends in surface water area, and CSV tables of historical annual time series of surface water areas. These files and their attributes are shown in Table 1. The Shapefiles are available as separate files for dry season, wet season and permanent water scenarios, and the area timeseries CSV table is a single file that includes all seasons. All these files are in ZIP archives. An accompanying README file concisely describes the attributes of each file.

**Table 1:** A description of the files and the attributes they contain, in the result data repository on Zenodo.

File names and description	Attribute names and description
**annualTrendsBasins_dry.zip** **annualTrendsBasins_permanent.zip** **annualTrendsBasins_wet.zip** **annualTrendsTransects_dry.zip** **annualTrendsTransects_permanent.zip** **annualTrendsTransects_wet.zip** Each of these is a ZIP archive of an ESRI Shapefile. They contain the estimated trends in annual surface water area from 1991 to 2020, for river basins and reaches (transects) in our study.	**HYBAS_ID** or **txId** (string): feature's unique identifier. - HYBAS_ID is for basins. It is the same as the basin's identifier HYBAS_ID in the HydroBASINS dataset - txId is for transects. It is the '_' concatenated string derived from the longitude and latitude values, truncated to 4 decimals, of the transect's centroid point. Specifically, it is “xx.xxxx_yy.yyyy” where xx.xxxx and yy.yyyy are the centroid's longitude and latitude values truncated to 4 decimals. **season** (string): Denotes the season. It is of the format “sss_mmm”. - “sss” denotes the season. “dry” is for dry season water, “wet” is for wet season water and “prm” is for permanent water. - “mmm” denotes the months the season spans (except for “permanent,” when this is “DnW” indicating dry and wet season). “fma” is for dry season of February-March-April, “ond” is for wet season of October-November-December. **sl_perYr** (float): Regression slope of the surface water area vs. year Sen's slope regression analysis. “_perYr” denotes its units, per year. The value -9999 indicates invalid trend, and occurs for cases where the trend was not estimated in our analysis because the feature was deemed to have inadequate valid data points in its area time series. **offset** (float): Regression offset of the surface water area vs. year Sen's slope regression analysis. NULL indicates invalid trend offset, and occurs where the trend was not estimated in our analysis because the feature was deemed to have inadequate valid data points in its area time series. **tsPtCount** (integer): Number of valid data-points in the area time series. Trend is estimated for features with this being 5 or greater.
**annualTimeseriesBasins.zip annualTimeseriesTransects.zip** Each of these is a ZIP archive of a CSV file. They contain the time series of surface water areas from 1991 to 2020 used for trend estimation, for the basins and reaches (transects) of our analysis.	**HYBAS_ID** or **txId** (string): feature's unique identifier. - HYBAS_ID is for basins. It is the same as the basin's identifier HYBAS_ID in the HydroBASINS dataset - txId is for transects. It is the ‘_’ concatenated string derived from the longitude and latitude values, truncated to 4 decimals, of the transect's centroid point. Specifically, it is “xx.xxxx_yy.yyyy” where xx.xxxx and yy.yyyy are the centroid's longitude and latitude values truncated to 4 decimals. **area_ha** (float): Feature's area in hectares **year** (float): Hydrologic year (June-May) **dry_fma_water_ha** (float): Area of water pixels in the feature in the dry season band of the annual composite image, in hectares. **dry_fma_nodata_ha** (float): Area of nodata pixels in the feature in the dry season band of the annual composite image, in hectares. **wet_ond_water_ha** (float): Area of water pixels in the feature in the wet season band of the annual composite image, in hectares. **wet_ond_nodata_ha** (float): Area of nodata pixels in the feature in the wet season band of the annual composite image, in hectares. **prm_DnW_water_ha** (float): Area of water pixels in the feature in the permanent band of the annual composite image, in hectares. **total_nodata_ha** (float): Area of nodata pixels in the feature in the permanent band of the annual composite image, in hectares.

The data stored within, and made accessible from, Google Earth Engine include the annual rate of change in SWA (in tabular format), timeseries of SWA (in tabular format), and annual seasonal composites with pixel-wise time-series of surface water occurrence (as raster layers) for each season. For each of these datasets, a Google Earth Engine cloud asset ID is provided for access to the data along with a description of data attributes.

### 3.3 Open methods

This section can be seen by selecting the ‘Open methods’ icon on the homepage or the ‘Methods’ tab on the Website header. This component is dedicated to describing the methods employed to generate this dataset, along with important limitations and caveats. Consequently, detailed information regarding this component is described in Section below.

## 4 Experimental Design, Materials and Methods

The surface water area trends dataset has been primarily derived from the Global Surface Water Explorer developed by the European Commission's Joint Research Centre (JRC), UN Environment and Google [2]. The JRC data maps terrestrial surface water globally using historical satellite imagery from the Landsat archive, at a spatial resolution of 30 m/pixel and a temporal resolution of once a month, from 1984 to 2021. This data is in raster format, where each pixel can have one of three values – No data (encoded as 0), Not water (encoded as 1), or water (encoded as 2).

Due to sparse data availability over India till 1990, our analysis begins with 1991. Consequently, this dataset provides users with a time-series of annual change in seasonal SWA (comprising wet-season, dry-season, and permanent seasons) from 1991 to 2020 for both river networks and sub-basins across India. All data processing and analyses have been conducted on Google Earth Engine [5].

**Fig 4:**
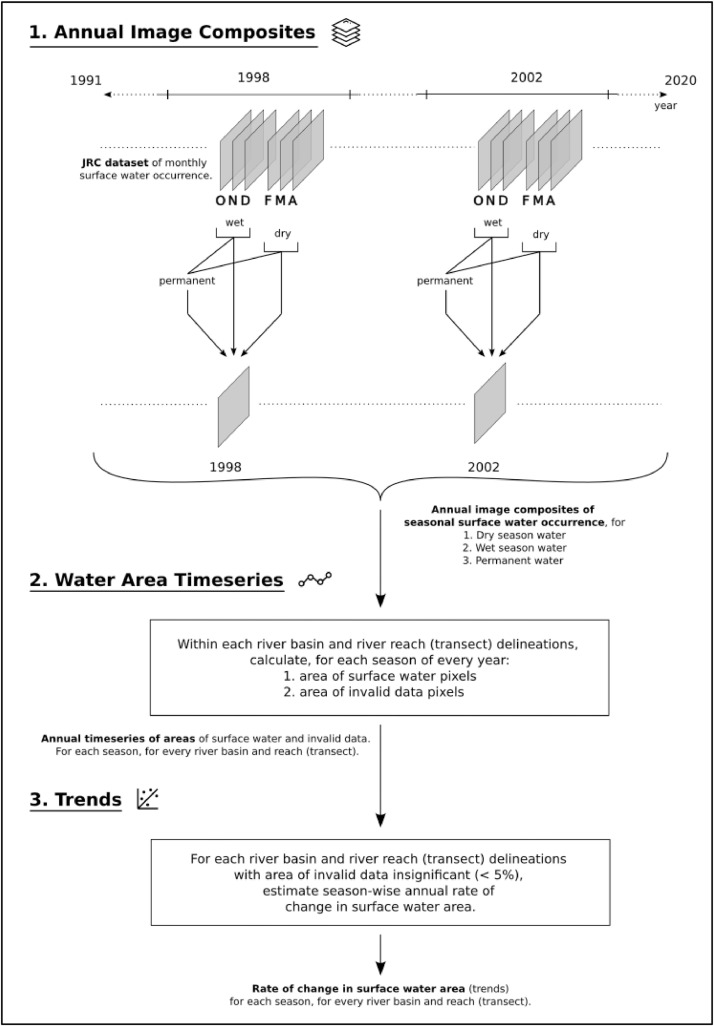
An overview of the workflow to generate the surface water area trends dataset, for river basins and reaches in India.

The main steps to generate the dataset include deriving seasonal and annual composites of surface water occurrence, generating a timeseries of SWA at the basin and reach scales, and conducting a trends analysis of annual change in SWA for each reach and basin. An overview of the workflow is illustrated in Fig. 4 and each step is expanded upon below.

### 4.1 Deriving seasonal and annual composites of surface water occurrence

The JRC data derived from Landsat satellite images, being prone to occlusion from cloud cover, is characterized by significant portions of missing data across spatiotemporal scales. Across India, the extent of missing or no data was particularly substantial for the monsoon months (June to September). Based on data completeness, we conducted our analysis across two seasons - the pre-monsoon or dry season comprising February, March and April, and the post-monsoon or wet season comprising October, November, and December. Combining data from these months for each year, we calculated water occurrence maps, called seasonal composites, for every year from 1991 to 2020, for dry and wet seasons separately. A combination of wet and dry seasonal composites further resulted in permanent water composites. For the purposes of our analysis, we counted a year ‘*y*’ to go from June of calendar year ‘*y*’ to May of calendar year ‘*y* + 1′. June is historically the start of monsoons for India, from which the country receives the bulk of its rain.

To create seasonal composites of SWA, the JRC data were similarly processed for transects and sub-basins. For each year, composites of wet and dry season SWA were calculated based on data aggregated at the scale of the pixel and season. At the pixel-level, every pixel in the JRC dataset has one of three values - water (2), not water (1), or no data (0) due to cloud cover or data inadequacy issues. Hence, a combination of three values corresponding to a 3-month season was available for every pixel. The **pixel-level aggregation** combined data for a three-month period to derive a seasonal composite of water (2), not water (1), or no data (0) as per the rules listed in Table 2.

**Table 2:** Rules for pixel-level data aggregation to derive seasonal composites.

Value of seasonal composite	Numerical representation	Compositing rules for water occurrence over a season (3 months) to drive a seasonal composite
No data	0	0 for all 3 months
Not water	1	1 in one or more months AND never 2
Water	2	2 in one or more months

For a given year, the **seasonal aggregation** combined the dry and wet season composites calculated above to derive annual seasonal composites of water for each pixel per year. Six annual composites were computed for each pixel and used for trend analysis – dry season water (all combinations that were water in the dry season, irrespective of the wet season composite), dry-season no-data, wet season water (all combinations that were water in the wet season, irrespective of the dry season composite), wet season no-data, permanent water (pixels that were water in both dry and wet seasons), and total no-data. The compositing rules used for the seasonal aggregation is shown in Table 3.

**Table 3:** Seasonal aggregation of wet and dry seasonal composites into dry season water, dry season no-data, wet season water, wet season no data, permanent water, and total no-data.

Annual Composite category	Permutations of wet & dry seasonal composites to derive annual composites (wet, dry composite values)				
Dry season Water	2,2	1,2	0,2		
Dry season No data	0,0	1,0	2,0		
Wet season Water	2,2	2,1	2,0		
Wet season No data	0,0	0,1	0,2		
Permanent Water	2,2				
Total No data	0,0	0,1	0,2	1,0	2,0

The compositing rules we define above are motivated by the goal of our analysis: to understand how surface water occurrence in India has changed at fine spatial scales over time by looking at these changes separately in the dry season, wet season, and permanent water scenarios. The rules for pixel-level aggregation (Table 2) were defined to maximize data validity and collect as much information about surface water occurrence. Consequently, our rule towards “no data” pixels was designed to be conservative: a pixel for a season is labelled as “no data” only when it is “no data” in all 3 months of the season. On the other hand, we had a more lenient requirement for valid water pixels: water occurring at a pixel in any of the 3 months, regardless of the other 2 months, is labelled as “water” for that season.

The rules for seasonal aggregation (Table 3) are straightforward for wet- and dry-season water: a year's pixel is labelled as wet/dry season water if it is water in that year's wet/dry seasonal composite respectively, regardless of value of the other season. Similarly, a year's pixel is taken to be dry season no-data if it is no-data in the year's dry season composite, regardless of the corresponding wet season composite. Likewise for wet season no-data. However, the rule for permanent water category is more conservative towards water occurrence; a pixel for a given year is designated as such only when it has water as its seasonal composite in both wet and dry seasons. A pixel's total no-data refers to when it is no-data in either or both the dry and wet season composites for that year. Overall, our chosen compositing rules, and consequently our area estimates and trend analysis that follow, tend to be generous towards counting water area in dry and wet seasons and conservative in counting area of permanent water.

### 4.2 Generating a timeseries of SWA at the basin and reach scale

Using our annual dry season water, wet season water and permanent water time series rasters at 30 m/pixel, we calculated water areas at river basin- and reach-scale for every year from 1991 to 2020. The time series of surface water areas that this yielded us enabled us to estimate their annual rates of changes at every one of our chosen basins and reaches.

We used the level 7 basins of the HydroBASINS dataset [4] as the basin layer within which SWA trends were analyzed. A total of 1516 sub-basins overlapped with India's national boundary. Of these, ∼176 sub-basins, largely within the Indian Himalayas and around India's borders, had inadequate valid data coverage due to satellite sensor issues, snow cover, and cloud cover, and were excluded from the analysis.

River reaches consist of rectangular transects spanning the cross-section of river flow lines. To create the reach layer, transect mid-points were laid roughly 1 km apart along every major river in India, using the HydroSHEDS flow accumulation data [4] at 15′' resolution. We define major rivers as those with a flow accumulation equal to or greater than 5000 cell counts, as pixels below this value typically represent river stretches too small to be faithfully captured given our resolution for analysis. We combined these transect mid-points with HydroSHEDS flow direction data [4] at 15" resolution, to create polygons perpendicular to the direction of flow at that location. Transects were constructed by combining blocks of 3 × 3 pixels of the JRC water dataset grid on either side of each transect's mid-point. Each such block of 3 × 3 JRC pixels creates an area of 90 m x 90 m, henceforth referred to as superpixels. To account for increasing river widths along a longitudinal gradient of a river, we examined a range of transect lengths across varying flow accumulation values. Based on a visual inspection, optimal reach sizes that would adequately span the cross section of conventional river reaches were identified (Table 4). Consequently, 4, 5 or 6 super-pixels were added to either side of the reach center point for different flow accumulation thresholds to construct each reach. Hence, each reach comprised a long and narrow polygon, within which SWA trends were analyzed. Although largely sufficient, reach lengths did not always span the entire cross section, particularly in cases such as along braiding river reaches, floodplains, and reservoirs. A total of 68,367 reaches were laid across India's river networks.

**Table 4:** Flow accumulation thresholds to determine transect size, and number of transects within each size category. A superpixel refers to a block of 3 × 3 pixels of the JRC dataset's pixel grid, and makes up an area of 90 m x 90 m. See Section 4.2 for more details.

Minimum and maximum thresholds of HydroSHEDS flow accumulation values	Transect size	Number of transects
5000 to 12,02,000	9 superpixels (72,900 m^2^)	66,198
12,02,000 to 32,69,000	11 superpixels (89,100 m^2^)	1789
Greater than 32,69,000	13 superpixels (1,05,300 m^2^)	380

A reach or basin for a given season and year was considered valid for trend analysis only if at least 95 % of its area contained valid data from the pixel-level aggregation.

### 4.3 Conducting a trends analysis of annual change in SWA

With the 30-years timeseries of annual surface water areas for every river basin and reach, we estimated the annual rate of change in surface water area using the Sen's slope linear trend estimator [3]. This method was selected since it provides a robust index of monotonic change and only requires the data to be independent without making any assumptions of normality of residual errors.

Trends in SWA were examined separately for permanent, wet-season, and dry-season surface water across every valid basin and reach (i.e., those with >95 % of valid data pixels). Seven categories or classes were used to visually represent the severity of change in SWA based on the Sen slope estimate, as indicated by the colored legend in the interactive map (Fig 1). In addition, the corresponding timeseries of no-data was also represented as a fractional area. These results are represented as a panel of figures in the Website as detailed in Section 3.1 (Fig 2). This corresponding timeseries of no-data is intended to help users gauge the certainty associated with reported surface water trends estimates. For years with low or nil proportions of invalid data, SWA estimates can be inferred to have high levels of certainty associated with them. Conversely, years with relatively higher proportions of missing data may be associated with lower levels of certainty.

To mitigate the risk of inferring patterns where there is very little valid data in the time series, we discarded those transects and sub-basins from the annual trend analysis that had fewer than 5 valid data points in their time series.

All the computer code that implements these methods on the Google Earth Engine are published in a public repository [6].

## Limitations

• Our annual image compositing rules are lenient towards dry and wet season water, while being strict towards permanent water. Consequently, our area estimates are relatively generous for dry and wet season water and conservative for permanent water.
• JRC historical surface water occurrence dataset [2] has `No data` pixels, indicating invalid data primarily due to snow or cloud cover, or unavailability of satellite image in the Landsat archive. Hence, some pixels in our annual image composites have invalid data, and basins or reaches with relatively large “no data” extents have been dropped from trend estimation.
• Surface water area dynamics are influenced by multiple factors (eg. anthropogenic disturbances, groundwater dynamics, seasons, land cover, etc.) in complex linear and non-linear ways, but here we estimate only a linear (monotonic) trend. We provide a time series dataset of surface water areas for users to undertake custom analyses to meet their specific objectives.
• Some rivers and water bodies could be missed, due to their small size relative to Landsat pixel resolution, or due to occlusion from covering or emerging vegetation
• Changes in surface water area due to transient events like floods and dam releases could be missed, since Landsat revisit cycle is typically 16 days.

## Ethics Statement

This work did not involve human subjects, animal experiments and data collected from social media platforms.

## CRediT authorship contribution statement

**Pradeep Koulgi:** Conceptualization, Methodology, Software, Validation, Formal analysis, Visualization, Data curation, Writing – original draft, Writing – review & editing. **Suman Jumani:** Conceptualization, Methodology, Validation, Writing – original draft, Writing – review & editing, Funding acquisition.

## Data Availability

Temporal trends of surface water area in India's rivers and basins (Original data) (Zenodo).
